# Global animal melioidosis prevalence: a systematic review and meta-analysis

**DOI:** 10.1186/s13620-026-00339-1

**Published:** 2026-03-24

**Authors:** Jongkonnee Thanasai, Atthaphong Phongphithakchai, Moragot Chatatikun, Sa-ngob Laklaeng, Jitbanjong Tangpong, Pakpoom Wongyikul, Phichayut Phinyo, Supphachoke Khemla, Anchalee Chittamma, Wiyada Kwanhian Klangbud

**Affiliations:** 1 Faculty of Medicine, Mahasarakham University, Mahasarakham, 44000 Thailand; 2 Nephrology Unit, Division of Internal Medicine, Faculty of Medicine, Prince of Songkla University, Songkhla, 90110 Thailand; 3 School of Allied Health Sciences, Walailak University, Nakhon Si Thammarat, 80160 Thailand; 4 Research Excellence Center for Innovation and Health Products (RECIHP), Walailak University, Nakhon Si Thammarat, 80160 Thailand; 5 Center for Clinical Epidemiology and Clinical Statistics, Faculty of Medicine, Chiang Mai University, Chiang Mai, 50200 Thailand; 6 Department of Biomedical Informatics and Clinical Epidemiology (BioCE), Faculty of Medicine, Chiang Mai University, Chiang Mai, 50200 Thailand; 7 Division of Infectious Diseases, Department of Internal Medicine, Nakhon Phanom Hospital, Nakhon Phanom, 48000 Thailand; 8 Department of Pathology, Faculty of Medicine Ramathibodi Hospital, Mahidol University, Bangkok, 10400 Thailand; 9 Medical Technology Program, Faculty of Science, Nakhon Phanom University, Nakhon Phanom, 48000 Thailand

**Keywords:** *Burkholderia pseudomallei*, Melioidosis, Animals, Prevalence, Systematic review, Meta-analysis, One Health

## Abstract

**Background:**

*Burkholderia pseudomallei*, the causative agent of melioidosis, infects both humans and a broad range of animal species. While human disease has been well characterized, animal data are dispersed across studies and lack a comprehensive global synthesis. This systematic review and meta-analysis estimated the pooled global prevalence of animal melioidosis and examined regional and diagnostic influences.

**Methods:**

Following PRISMA 2020 guidelines, studies reporting *B. pseudomallei* detection in animals were retrieved from PubMed, Scopus, and Embase up to April 2025. Pooled prevalence was calculated using a random-effects model. Subgroup and meta-regression analyses explored moderators of heterogeneity.

**Results:**

Twenty-eight studies from 11 countries, encompassing 98,885 animal samples, were included. The global pooled prevalence was 7.3% (95% CI: 0.039−0.117), with high heterogeneity (*I*² = 98.8%). Southeast Asia, particularly Thailand, showed consistently higher prevalence across livestock, wildlife, and companion animals. Diagnostic methods and study period contributed to variability but were not statistically significant.

**Conclusions:**

Animal melioidosis is globally distributed, with Thailand serving as a key endemic region and research hub. Strengthening One Health surveillance, standardizing diagnostic tools, and expanding genomic monitoring are essential for improved detection and control of *B. pseudomallei* across animal populations.

**Supplementary Information:**

The online version contains supplementary material available at 10.1186/s13620-026-00339-1.

## Background

Melioidosis, caused by *Burkholderia pseudomallei*, is an emerging infectious disease that predominantly affects individuals in tropical and subtropical regions. It is associated with a range of severe symptoms, including sepsis, pneumonia, and abscess formation, leading to high morbidity and mortality, especially in countries such as Thailand, Australia, and Southeast Asia [1]. Beyond its clinical severity, melioidosis also places a significant economic burden on endemic regions due to prolonged hospitalization, intensive antimicrobial therapy, and productivity losses in both humans and livestock [1, 2]. Although substantial research has addressed the global distribution, clinical impact, and antimicrobial resistance of human melioidosis [1–3], comparatively little attention has been given to animal infections and their epidemiological significance. Animals, including livestock, companion animals, and wildlife, serve as potential reservoirs for *B. pseudomallei* and can contribute to the zoonotic transmission of the disease [2]. The increasing threat of climate change, which is predicted to alter the distribution of B. *pseudomallei*, further underscores the need to understand the disease’s dynamics not just in humans but in animals as well [4]. However, despite these alarming trends, data on animal melioidosis remains sparse, and systematic data on the disease in animals are fragmented, hindering effective surveillance and control strategies.

Recent studies have identified critical gaps in the current understanding of melioidosis. For instance, the study by Thanasai et al. (2025) examined antibiotic resistance in *B. pseudomallei* isolates from human clinical cases and revealed significant regional variations in resistance rates, but animal infections were not included in their analysis [3]. This gap highlights the need for better surveillance that integrates both human and animal health data. Furthermore, Birnie et al. (2022) reviewed the epidemiological transitions of melioidosis, noting that while the disease is becoming increasingly recognized in non-endemic regions [2], comprehensive data on animal melioidosis remain lacking. This absence of data prevents an accurate understanding of the full burden of the disease, particularly in newly emerging regions. Similarly, Thanasai et al. (2025) noted discrepancies in diagnostic methods used across studies, making cross-study comparisons difficult, and underscoring the need for standardized diagnostic frameworks [3]. Additionally, Li et al. (2025) demonstrated that climate change could expand the risk of melioidosis, but the role of animals in these emerging risk zones was not fully explored [4], pointing to another key research gap.

This study seeks to address these gaps by conducting a comprehensive systematic review and meta-analysis of the global prevalence of animal melioidosis. By standardizing diagnostic methods, the study will estimate the pooled prevalence of *B. pseudomallei* infections across species and regions, providing much-needed insights into regional hotspots and the influence of diagnostic techniques. Furthermore, this research will explore the ecological and epidemiological factors influencing the transmission dynamics of *B. pseudomallei*, particularly in the context of climate change. The study’s findings will contribute to global surveillance efforts, strengthen One Health frameworks, and offer recommendations for improving early detection and intervention strategies to reduce the zoonotic risks of melioidosis [5, 6].

## Methods

### Protocol Registration

This systematic review and meta-analysis was conducted following the Preferred Reporting Items for Systematic Reviews and Meta-Analyses (PRISMA) 2020 guidelines [7]. The review protocol was prospectively registered in the PROSPERO International Prospective Register of Systematic Reviews (Registration No. CRD420251161197). The protocol defined the review question, inclusion criteria, data extraction procedures, and quality appraisal methods used to ensure transparency and reproducibility.

### Search strategy

Following PRISMA 2020 guidelines, we conducted comprehensive database searches in PubMed, Scopus, and Embase up to April 2025. The search strings were constructed around three core components—pathogen (*Burkholderia pseudomallei* OR melioidosis), host (animal OR livestock OR wildlife OR veterinary), and epidemiological outcome (prevalence OR seroprevalence OR infection)—combined using Boolean operators. Each database used its own controlled vocabulary (e.g., MeSH in PubMed, Emtree in Embase) and field tags; the complete strategies are summarized in Supplementary Table S1.

### Eligibility criteria

Studies were eligible if they: (1) Reported primary data on detection or isolation of *B. pseudomallei* in any animal species; (2) Provided sufficient quantitative information to calculate prevalence; (3) Used laboratory-based diagnostic methods (serological methods [IHA; indirect hemagglutination, CFT; complement fixation test, or ELISA; enzyme linked immunosorbent assay], molecular methods [PCR; polymerase chain reaction, qPCR; quantitative PCR, WGS [whole genome sequencing], or culture or mixed). Exclusion criteria included: narrative or systematic reviews, conference abstracts, experimental infections, and reports without extractable prevalence data.

### Data extraction

Two independent reviewers extracted study characteristics using a standardized form, including: author, year, country, and region; animal species and host group (livestock, wildlife, companion); diagnostic method(s); number of animals tested and positive; setting (farm, abattoir, zoo, field survey). Discrepancies were resolved by discussion. Extracted data were compiled in Microsoft Excel 2021 for meta-analysis.

### Quality assessment

The methodological quality of the included studies was evaluated using the Joanna Briggs Institute (JBI) Critical Appraisal Checklist for Studies Reporting Prevalence Data. This nine-item tool assesses sampling adequacy, representativeness, diagnostic validity, data analysis, and precision of prevalence estimates. Each criterion was scored as “Yes” (1), “No” (0), or “Unclear” (0.5), giving a total score out of nine. Studies were classified as high quality (8–9 points), moderate quality (6–7 points), or low quality (≤ 5 points). Two reviewers independently assessed all 27 studies reporting the prevalence of *Burkholderia pseudomallei* in animals from 1970 to 2025, covering multiple countries, species, and diagnostic methods. Discrepancies were resolved by consensus. A full summary of the appraisal, including individual study scores, strengths, and limitations, is provided in Supplementary Table S2.

### Statistical analysis

All analyses were conducted using R software (version 4.3.1) with the meta and *metafor* packages. The pooled prevalence of *Burkholderia pseudomallei* infection was calculated using a random-effects model (DerSimonian–Laird method) after applying the Freeman–Tukey double arcsine transformation to stabilize variance. Heterogeneity was assessed using the *I*² and τ² statistics, with *I*² values > 75% indicating substantial heterogeneity.

Subgroup analyses were performed by animal group, region, diagnostic method, and study period. Meta-regression was used to explore potential moderators, including region, diagnostic approach, and publication year. Sensitivity analysis assessed the influence of individual studies on the pooled estimate. Publication bias was evaluated visually by funnel plot and statistically using Egger’s regression test. A *p*-value < 0.05 was considered significant.

To visually assess the relationship between study size and observed prevalence, a bubble plot was generated using sample size as the x-axis (log-transformed), observed prevalence as the y-axis, and bubble size proportional to study sample size. This graphical approach was used to explore potential small-study effects and sources of heterogeneity.

## Results

### Study selection

**Fig. 1:**
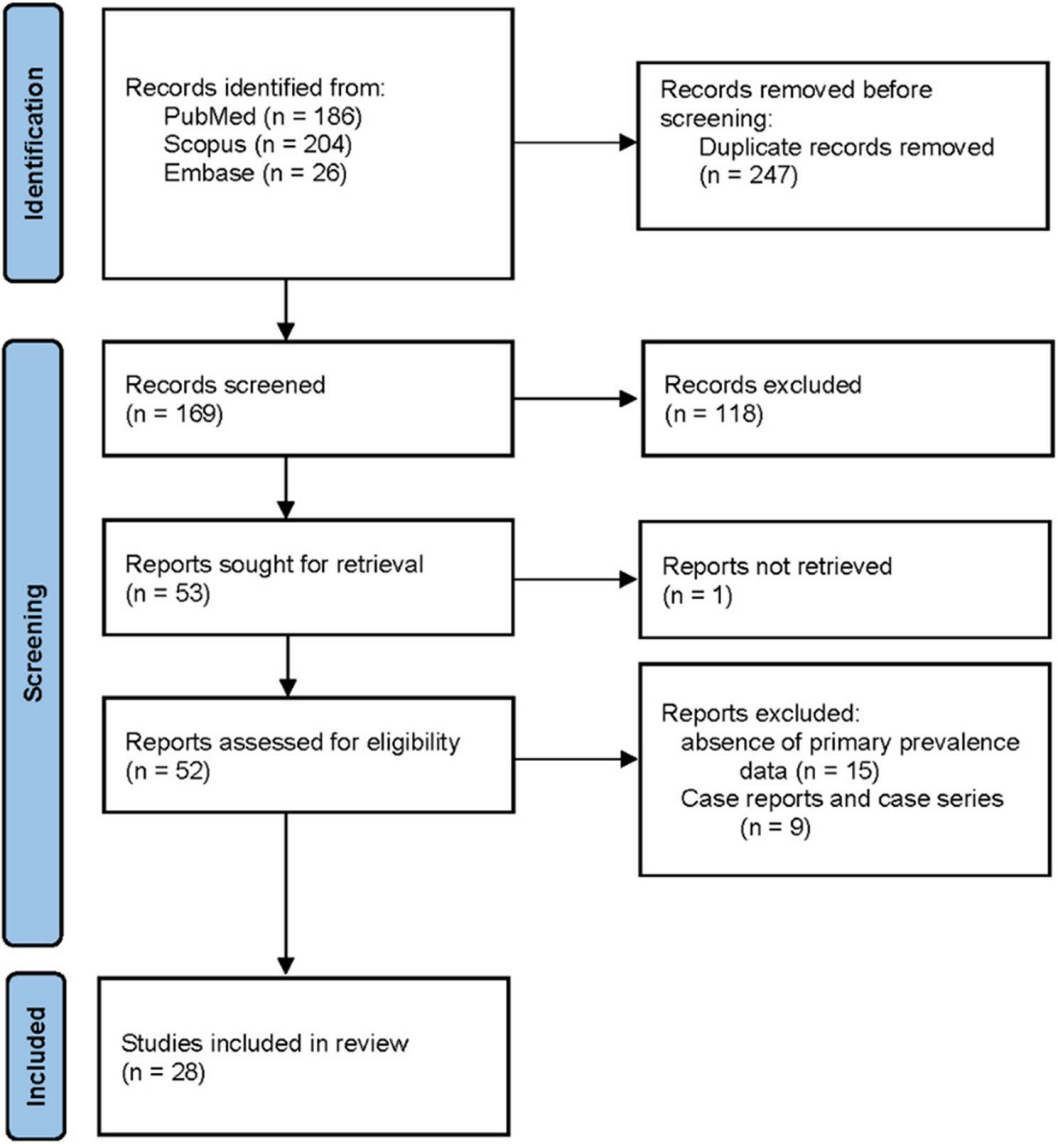
PRISMA flow diagram

A total of 416 records were identified through database searches (PubMed = 186, Scopus = 204, Embase = 26). After removing 247 duplicates, 169 unique records were screened by title and abstract. Fifty-two full-text articles were retrieved and assessed for eligibility, of which 28 studies met the inclusion criteria for both qualitative and quantitative synthesis. Reasons for exclusion included absence of primary prevalence data (*n* = 15), and case reports and case series (*n* = 9). The study selection process is illustrated in Fig. 1 (PRISMA flow diagram).

### Characteristics of included studies

The 28 included studies [8−35], published between 1970 and 2025, spanned 11 countries across five global regions (Southeast Asia, Oceania, Africa, the Middle East, and North America). Combined, they analyzed 98,885 animal samples, representing diverse hosts including cattle, pigs, goats, sheep, horses, wallabies, birds, and reptiles.

Most studies were conducted in Southeast Asia (*n* = 16), mainly Thailand, which contributes the majority of data [9–11, 13, 20, 24, 26–28, 33, 34]. Diagnostic techniques included culture isolation (*n* = 5), serological assays (*n* = 18), molecular methods (*n* = 1), and mixed (more than one method; *n* = 4). A detailed summary of the included studies is presented in Table 1.

**Table 1:** Characteristics of included studies

**Author, Year**	**[Ref]**	**Country**	**Region**	**Animal Group**	**Specie**	**Diagnostic Method**	**N (98,632)**	**Positive**	**Prevalence (%)**
Alexander et al., 1972	[8]	Vietnam	Southeast Asia	Companion	Dogs	IHA	64	12	18.75
Bunterm et al., 2013 (Th)	[9]	Thailand	Southeast Asia	Livestock	Cattle	IHA	4126	165	4.00
Choldumrongkul et al., 2005 (Th)	[10]	Thailand	Southeast Asia	Livestock	Goats	IHA	191	19	9.95
Damrongsukij et al., 2021	[11]	Thailand	Southeast Asia	Wildlife	Macaques	IHA	28	16	57.14
Ekakoro et al., 2022	[12]	Uganda	Africa	Livestock	Pigs	ELISA	1035	93	8.99
Fungwithaya et al., 2024	[13]	Thailand	Southeast Asia	Companion	Dogs	IHA	156	9	5.77
Gasqué et al., 2024a	[14]	Guadeloupe	North America	Livestock	Goats	PCR, ELISA	31	12	38.71
Gasqué et al., 2024b	[15]	Guadeloupe, French Guiana	North America	Livestock	Goats, Sheep, Equids, Pigs	ELISA	701	91	12.98
Hambali et al., 2018	[16]	Malaysia	Southeast Asia	Livestock	Small ruminants	CFT	200	1	0.50
Hampton et al., 2011	[17]	Australia	Oceania	Wildlife	Birds	Culture	110	1	0.91
Hemme et al., 2016	[18]	Puerto Rico	North America	Wildlife	Non-human primates	IHA	24	1	4.17
Höger et al., 2016	[19]	Australia	Oceania	Mixed	Multi species	Culture, PCR	357	7	1.96
Jeenpun et al., 2013 (Th)	[20]	Thailand	Southeast Asia	Mixed	Zoo animals, Reptiles, Birds	IHA	65	1	1.54
Kaufmann et al., 1970	[21]	USA	North America	Wildlife	Macaques	IHA	284	11	3.87
Mosavari et al., 2024	[22]	Iran	Middle East	Livestock	Sheep, Goats, Cattle	PCR	97	0	0.00
Musa et al., 2023	[23]	Malaysia	Southeast Asia	Livestock	Livestock	CFT	72941	4516	6.19
Naksuwan et al., 2023 (Th)	[24]	Thailand	Southeast Asia	Livestock	Goats	Culture	223	41	18.39
Norris et al., 2020	[25]	Vietnam	Southeast Asia	Livestock	Pigs	ELISA	1125	71	6.31
Punchoopet et al., 2012 (Th)	[26]	Thailand	Southeast Asia	Wildlife	Elephants	IHA	485	123	25.36
Saechan et al., 2022	[27]	Thailand	Southeast Asia	Wildlife	Macaques	IHA	223	32	14.35
Srikitjakarn, et al., 2002	[28]	Thailand	Southeast Asia	Livestock	Cattle	IHA	253	5	1.97
Taetzsch et al., 2022	[29]	USA	North America	Wildlife	Macaques	Culture, PCR, WGS	360	1	0.28
Thomas et al., 1981a	[30]	Australia	Oceania	Mixed	Multi species	Culture	220	48	21.82
Thomas et al., 1981b	[31]	Australia	Oceania	Livestock	Pigs	Culture	7908	14	0.18
Thomas et al., 1988	[32]	Australia	Oceania	Livestock	Goats	Culture, IHA, CFT	3143	112	3.56
Tongnoon et al., 2004 (Th)	[33]	Thailand	Southeast Asia	Livestock	Goats	IHA	3546	14	0.39
Tonpitak et al., 2014	[34]	Thailand	Southeast Asia	Livestock	Goats	Culture	72	10	13.89
Zheng et al., 2025	[35]	Lao PDR	Southeast Asia	Livestock	Pigs, Cattle, Buffalo	IHA	917	111	12.10

*IHA* Indirect hemagglutination, *CFT* Complement fixation test, *ELISA* Enzyme-linked immunosorbent assay, *PCR* Polymerase chain reaction,* qPCR* quantitative PCR, *WGS* Whole genome sequencing, *Th* Thai language, *PDR *People’s Democratic Republic

### Quality of included studies

Overall methodological quality was high across the included studies. Of the 28 papers, 24 (85.77%) were rated as high quality and 4 (14.3%) as moderate quality; none were low quality. Recent studies (post-2010) generally demonstrated comprehensive sampling, validated diagnostics, and appropriate statistical analyses, whereas older reports were limited by small sample sizes or incomplete reporting.

Methodological quality has improved over time, reflecting greater epidemiological rigor and the adoption of standardized diagnostic methods. All studies were retained for meta-analysis, with weights applied based on JBI quality scores. The detailed JBI checklist and results are presented in Supplementary Table S2.

### Global pooled prevalence of burkholderia pseudomallei

The random-effects meta-analysis estimated a global pooled prevalence of 7.3% (95% CI: 0.039−0.117) for *B. pseudomallei* infection or exposure among animals. Individual study estimates ranged widely, from 0 to over 57.1%, reflecting diverse ecological conditions, diagnostic methods, and animal populations.

**Fig. 2:**
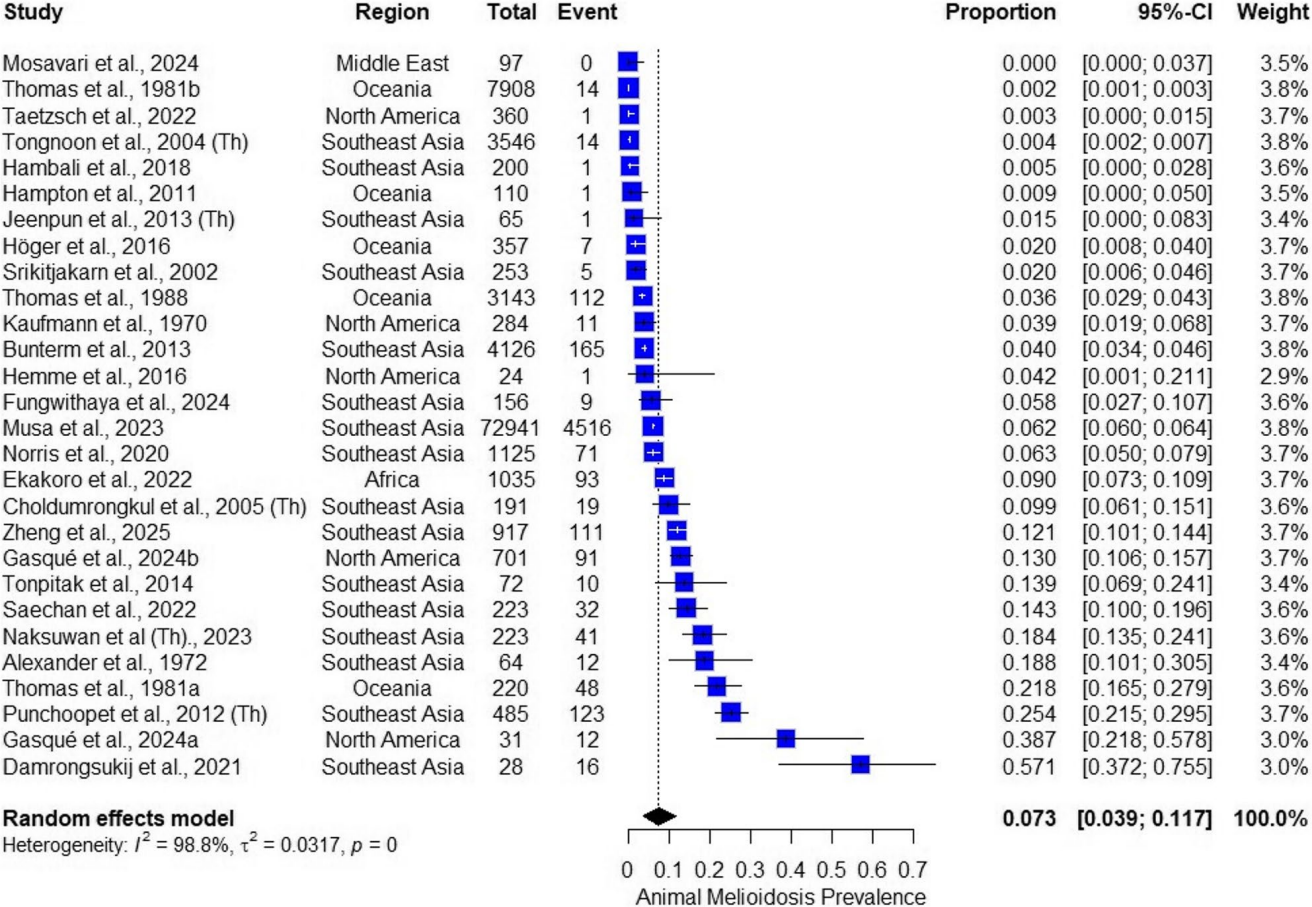
Forest plot of overall pooled prevalence

Substantial heterogeneity was observed (*I*² = 98.8%, τ² = 0.0317, *p* = 0), indicating significant between-study variability. The forest plot (Fig. 2) depicts individual study prevalence with corresponding 95% confidence intervals and the overall pooled estimate. Higher prevalence was notably observed in studies conducted in northern Australia, Thailand, and Malaysia, consistent with recognized endemic regions.

**Fig. 3:**
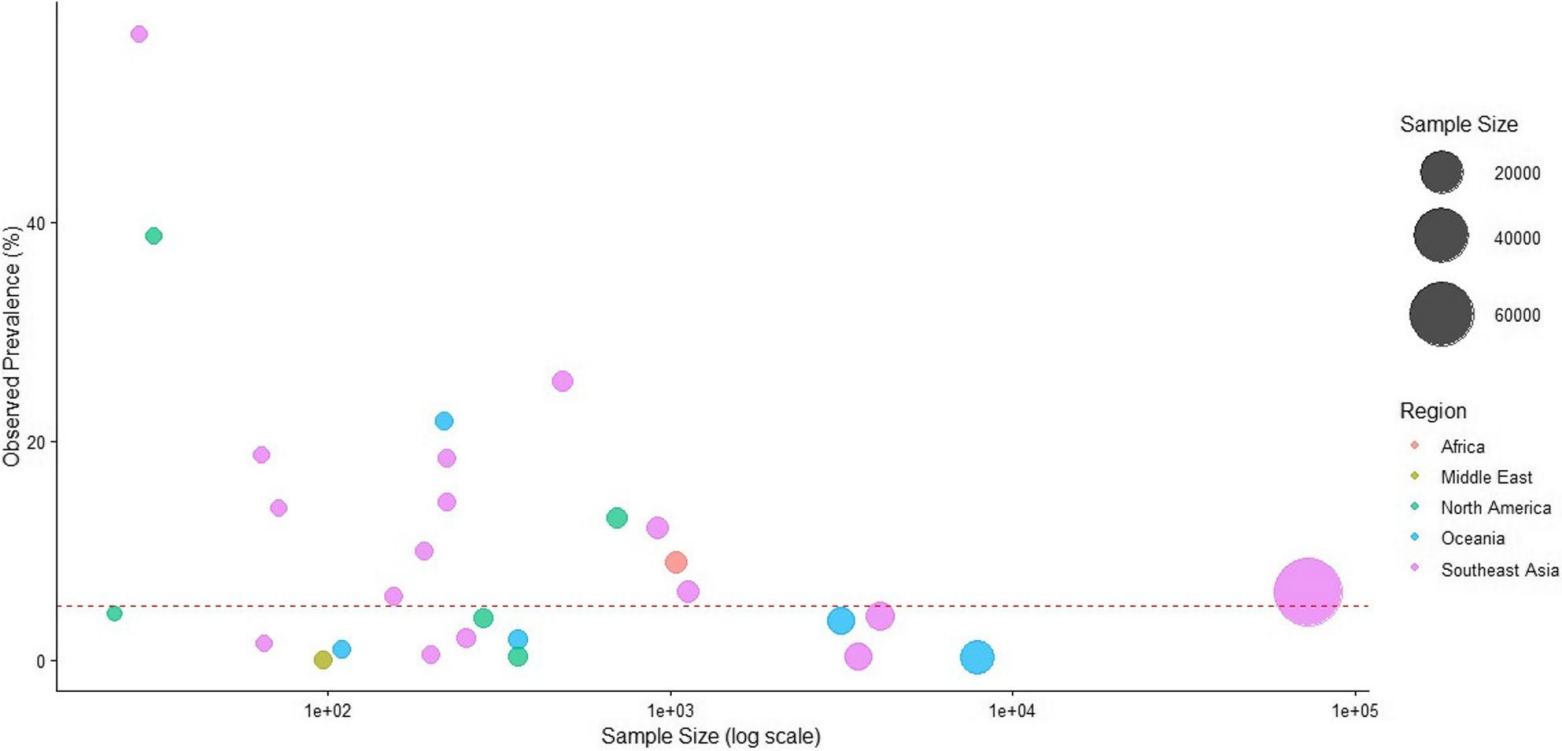
Bubble plot of animal melioidosis prevalence

To further examine variability across studies, a bubble plot was generated (Fig. 3). The plot shows greater dispersion and more extreme prevalence values in smaller studies, whereas larger studies tend to cluster around the pooled estimate. This pattern suggests that sampling variability and localized ecological conditions likely contribute to the observed heterogeneity rather than systematic bias.

### Subgroup analyses

#### By animal group

Subgroup analysis by animal category showed variations in prevalence (Table 2 and Supplementary Figure S1). Companion had the highest pooled prevalence of 11.1% (95% CI: 0.000−1.000), wildlife showed 10.3% (95% CI: 0.002−0.303), mixed showed 6.4% (95% CI: 0.000−0.467), and livestock animals exhibited 5.8% (95% CI: 0.025−0.104). While companion populations demonstrated higher infection rates, statistical comparison revealed no significant difference among animal groups (*p* = 0.7396). These findings suggest that environmental exposure, rather than animal taxonomy, may drive infection risk.

**Table 2:** Summary of pooled prevalence estimates across key subgroups

Category	Subgroup	Studies (*n*)	Pooled Prevalence (%)	95% CI	I² (%)	*p*-value Between-group
**Animal Group**	Livestock	16	5.8	0.025−0.104	99.2	0.7396
	Wildlife	6	10.3	0.002−0.303	97.6	
	Companion	2	11.1	0.000−1.000	87.0	
	Mixed	3	6.4	0.000−0.467	97.0	
**Region**	Southeast Asia	16	8.7	0.040−0.149	98.3	< 0.0001
	North America	5	8.1	0.000−0.297	96.5	
	Oceania	5	3.6	0.000−0.155	98.8	
	Africa	1	9.0	0.059−0.149	-	
	Middle East	1	0.0	0.000−0.037	-	
**Diagnostic Method**	Serology	18	7.7	0.038−0.128	98.1	0.0019
	Culture	5	8.2	0.000−0.316	94.4	
	Molecular	1	0.0	0.000−0.270	-	
	Mixed	4	4.4	0.000−0.316	94.4	
**Study Period**	Pre-2000	5	6.7	0.000−0.233	99.0	0.4172
	2000–2010	3	2.9	0.000−0.233	96.3	
	2011–2020	9	5.0	0.011−0.113	96.5	
	2021–2025	11	10.3	0.033−0.204	96.3	

#### By geographic region

Regional differences in prevalence were also evaluated (Table 2 and Supplementary Figure S2). The highest pooled prevalence was recorded in Africa (9.0%, 95% CI: 0.059−0.096), and the lowest was in the Middle East (0.0%, 95% CI: 0.000−0.037). However, they were only one study in each region. For groups of more than one study, the highest was in Southeast Asia (8.7%), followed by North America (8.1%) and Oceania (3.6%). There are significant differences between geographic regions (*p* < 0.0001). The high rates in Southeast Asia are associated with endemic environmental reservoirs of *B. pseudomallei* in tropical zones.

#### By diagnostic method

When stratified by diagnostic approach, pooled prevalence (Table 2 and Supplementary Figure S3), serological methods exhibited 7.0% (95% CI: 0.037−0.111), culture method was 8.2% (95% CI: 0.000−0.270), mixed methods showed 4.4% (95% CI: 0.000−0.316), and molecular methods showed 0.0% (95% CI: 0.000−0.037). As shown in Fig. 5, culture-confirmed studies generally produced higher prevalence with a higher specificity. Moreover, subgroup differences between diagnostic methods were statistically significant (*p* = 0.0019).

#### By study period

Temporal subgroup analysis across four intervals (pre-2000, 2000–2010, 2011–2020, and 2021–2025) demonstrated a gradual but non-significant increase in prevalence (Table 2 and Supplementary Figure S4). Pre-2000 was 6.7% (95% CI: 0.000−0.230), 2000–2010 was 2.9% (95% CI: 0.000−0.233), 2011–2020 was 5.0% (95% CI: 0.0011−0.113), and 2021–2025 was 10.3% (95% CI: 0.033−0.204). The upward trend likely reflects improved diagnostic sensitivity and enhanced surveillance efforts rather than a genuine epidemiological surge. Statistical testing showed no significant temporal effect (*p* = 0.4172).

#### Meta-regression analyses

Meta-regression was performed to examine whether animal group, geographic region, diagnostic method, or study period explained the high heterogeneity observed across studies (Supplementary File S1). None of these variables significantly influenced prevalence estimates: region (*p* = 0.574), animal group (*p* = 0.797), diagnostic method (*p* = 0.660), and study period (*p* = 0.409).

In all models, the proportion of heterogeneity explained was negligible (R² = 0%), and residual heterogeneity remained very high (*I*² approximately 99%, all *p* < 0.001). These results indicate that the variability between studies was not explained by the examined subgroup factors.

#### Publication bias

Visual inspection of the funnel plot did not indicate substantial asymmetry, with study estimates distributed relatively evenly around the pooled effect (Supplementary Figure S5). Egger’s regression test further supported the absence of small-study effects (*t* = 0.12, *p* = 0.9029). Together, these findings suggest no statistically significant evidence of publication bias in the present meta-analysis.

## Discussion

This systematic review and meta-analysis provide the most comprehensive synthesis to date of *Burkholderia pseudomallei* infection among global animal populations. Drawing upon five decades of research from 11 countries and nearly 100,000 animal samples, this study establishes that *B. pseudomallei* infection is not confined to particular species or regions but is instead an ecologically entrenched zoonotic threat with a wide environmental range. The pooled global prevalence of 7.3% (95% CI: 0.039−0.117) underscores a moderate but persistent burden of infection, especially in tropical and subtropical zones where the pathogen is endemic, such as Southeast Asia and northern Australia. These results reaffirm that melioidosis remains a neglected but globally relevant zoonosis influenced by environmental and anthropogenic changes [1, 4].

Thailand contributed the largest body of evidence among all included countries, with multiple high-quality investigations providing valuable insight into the epidemiology of animal melioidosis across host species and ecological zones. For instance, Bunterm et al. [9] identified a 4.0% seroprevalence in dairy cattle from Chaiyaphum Province, emphasizing the ongoing exposure risk in northeastern Thailand, an area known for intensive livestock farming. Similarly, Choldumrongkul et al. [10] reported a 9.9% infection rate among goats in southern provinces, revealing localized clusters likely driven by soil and water contamination. Studies in zoo and wildlife settings, such as Jeenpun et al. [20] and Punchoopet et al. [26], further highlighted cross-species vulnerability: elephants and zoo animals exhibited seropositivity rates of 25% and 1.5%, respectively, suggesting that *B. pseudomallei* can persist even in controlled or semi-urban environments.

More recent Thai studies have expanded the understanding of melioidosis epidemiology. Naksuwan et al. [24] reported an 18.4% prevalence in goats from western Thailand between 2007 and 2016, linking outbreaks to climatic fluctuations and wet-season exposure. Tonpitak et al. [34] documented a fatal melioidosis outbreak in goats in Bangkok, demonstrating that the disease can extend into peri-urban agricultural systems and pose risks to both animal handlers and nearby human populations. Likewise, Thongnoon et al. [33] detected low-level seropositivity (0.4%) in goats in Thailand’s southern border provinces, possibly indicating subclinical or past infections in regions with less environmental exposure. Collectively, these findings reinforce that melioidosis is endemic across multiple Thai ecological settings, from rural livestock farms to urban peripheries, and that both exposure intensity and diagnostic capacity influence reported prevalence.

Diagnostic variability remains a major determinant of heterogeneity across studies. Serological techniques such as indirect hemagglutination (IHA) and ELISA generally yielded higher prevalence estimates due to their ability to detect previous exposure and potential cross-reactivity with closely related *Burkholderia* species. In contrast, culture-based and molecular diagnostics, although highly specific, often underreport infection due to bacterial fastidiousness, sample degradation, or laboratory limitations in endemic areas. This discrepancy was evident in several Thai investigations where serological surveys [10, 11, 13, 20, 26–28, 33] indicated substantial exposure despite the limited number of culture-confirmed cases [24, 34]. The development of standardized, validated diagnostic frameworks is thus essential to enable meaningful interstudy and interregional comparisons.

Although meta-regression did not reveal statistically significant temporal trends, a gradual increase in reported prevalence was observed from pre-2000 to 2025. This trend likely reflects improvements in diagnostic sensitivity, greater research activity, and enhanced disease awareness rather than a true rise in global incidence. Notably, the expanding recognition of melioidosis in temperate or non-endemic regions, such as North America and the Caribbean [14, 15, 18, 21, 29], suggests a widening ecological niche. Climate change, by modifying rainfall patterns, soil moisture, and temperature, may facilitate northward and altitudinal shifts of *B. pseudomallei* distribution [3]. The substantial prevalence reported from Thai and Malaysian livestock [9, 10, 16, 23, 24, 28, 33] further underscores Southeast Asia’s vulnerability to environmental drivers, particularly during monsoonal flooding events that enhance bacterial dispersion and animal exposure. Similarly, Zheng et al. (2025) reported high seroprevalence among cattle (up to 22.8%) and buffaloes (up to 70.6%) in Lao People’s Democratic Republic [35], reinforcing the presence of widespread livestock exposure in neighboring endemic settings.

From a One Health perspective, animal melioidosis serves not only as a veterinary issue but also as a sentinel indicator of environmental contamination and zoonotic potential. The detection of *B. pseudomallei* across livestock, companion, and wildlife species in Thailand and neighboring countries demonstrates the interconnectedness of environmental, animal, and human health systems. In Thailand, where both veterinary and public health surveillance infrastructure are relatively advanced, these studies exemplify how integrated monitoring can illuminate transmission pathways and inform targeted interventions.

However, diagnostic capacity and coordinated reporting remain limited in many endemic settings. Integrating animal surveillance data into national melioidosis registries—currently focused primarily on human cases—would enhance early outbreak detection and risk assessment. Additionally, genomic epidemiology offers promising opportunities for elucidating cross-species transmission dynamics. Whole-genome sequencing (WGS) has already revealed shared *B. pseudomallei* sequence types between humans, animals, and environmental isolates [1], yet few Thai or regional animal studies have applied this approach. Expanding WGS-based surveillance will be vital to track virulent strains, monitor antimicrobial resistance, and assess environmental persistence.

Several limitations should be acknowledged. First, substantial heterogeneity was observed across studies, indicating considerable variability beyond chance alone. Although subgroup and meta-regression analyses were conducted, the examined moderators did not significantly explain this heterogeneity, suggesting that unmeasured ecological, environmental, and methodological factors may have influenced prevalence estimates. Second, diagnostic variability across studies likely contributed to inconsistencies in reported prevalence. Most included studies relied on serological methods such as IHA or ELISA, which may detect prior exposure rather than active infection and may be subject to cross-reactivity. In contrast, culture and molecular methods, while more specific, may underestimate prevalence due to sampling and laboratory constraints. Third, the majority of included studies originated from Southeast Asia, particularly Thailand, potentially limiting the generalizability of the findings to underrepresented regions. Data from Africa, the Middle East, and parts of the Americas were limited and often based on single studies. Finally, the absence of standardized animal surveillance systems and harmonized diagnostic frameworks across countries restricts direct comparability between studies. Differences in sampling strategies, case definitions, and laboratory capacity may have influenced reported prevalence estimates.

## Conclusion

This systematic review and meta-analysis reveal a global pooled prevalence of 4.9% for *Burkholderia pseudomallei* infection in animals, confirming its widespread and persistent presence across tropical and subtropical regions. Southeast Asia, especially Thailand, contributes the majority of data, with several Thai studies [9–11, 13–20, 24, 27, 28, 33] reporting endemic infections in livestock, wildlife, and companion animals. These findings highlight Thailand’s pivotal role in regional melioidosis surveillance.

Environmental factors, rather than host species, primarily drive infection risk, while variability in diagnostic methods underscores the need for standardized, validated assays. Integrating molecular and genomic surveillance into One Health frameworks is essential to improve detection and trace cross-species transmission.

In summary, *B. pseudomallei* remains a neglected but significant zoonosis. Strengthening One Health surveillance, harmonizing diagnostic standards, and expanding genomic monitoring will be critical to control animal melioidosis and mitigate zoonotic risks worldwide.

## Supplementary Information

Supplementary Material 1.

Supplementary Material 2.

Supplementary Material 3.

Supplementary Material 4.

Supplementary Material 5.

Supplementary Material 6.

Supplementary Material 7.

Supplementary Material 8.

## Data Availability

No datasets were generated or analysed during the current study.

## Abbreviations

CI: Confidence interval
CFT: Complement fixation test
ELISA: Enzyme-linked immunosorbent assay
IHA: Indirect hemagglutination
PCR: Polymerase chain reaction
PRISMA: Preferred Reporting Items for Systematic Reviews and Meta-Analyses
qPCR: Quantitative PCR
Th: Thai language
WGS: Whole genome sequencing
