# Supplementary material for: Global animal melioidosis prevalence: a systematic review and meta-analysis
Source: Ir Vet J. 2026 Mar 24;79:26. doi: 10.1186/s13620-026-00339-1 (PMC13134086; doi:10.1186/s13620-026-00339-1)

**Global Animal Melioidosis Prevalence: A Systematic Review and Meta-analysis**

Jongkonnee Thanasai^1^, Atthaphong Phongphithakchai^2^, Moragot Chatatikun^3,4^, Sa-ngob Laklaeng^3^, Jitbanjong Tangpong^3,4^, Pakpoom Wongyikul^5,6^, Phichayut Phinyo^5,6^, Supphachoke Khemla^7^, Anchalee Chittamma^8^, Wiyada Kwanhian Klangbud^9,*^

^1^ Faculty of Medicine, Mahasarakham University, Mahasarakham 44000, Thailand

^2^ Nephrology Unit, Division of Internal Medicine, Faculty of Medicine, Prince of Songkla University, Songkhla 90110, Thailand

^3^ School of Allied Health Sciences, Walailak University, Nakhon Si Thammarat 80160, Thailand

^4^ Research Excellence Center for Innovation and Health Products (RECIHP), Walailak University, Nakhon Si Thammarat 80160, Thailand

^5^  Center for Clinical Epidemiology and Clinical Statistics, Faculty of Medicine, Chiang Mai University, Chiang Mai 50200, Thailand

^6^ Department of Biomedical Informatics and Clinical Epidemiology (BioCE), Faculty of Medicine, Chiang Mai University, Chiang Mai 50200, Thailand

^7^ Division of Infectious Diseases, Department of Internal Medicine, Nakhon Phanom Hospital, Nakhon Phanom 48000, Thailand

^8^ Department of Pathology, Faculty of Medicine Ramathibodi Hospital, Mahidol University, Bangkok 10400, Thailand

^9^ Medical Technology Program, Faculty of Science, Nakhon Phanom University, Nakhon Phanom 48000, Thailand

***** Corresponding author: Wiyada Kwanhian Klangbud, wiyadakwanhian@gmail.com

**Supplementary Figure S2.** Forest plot by animal group.


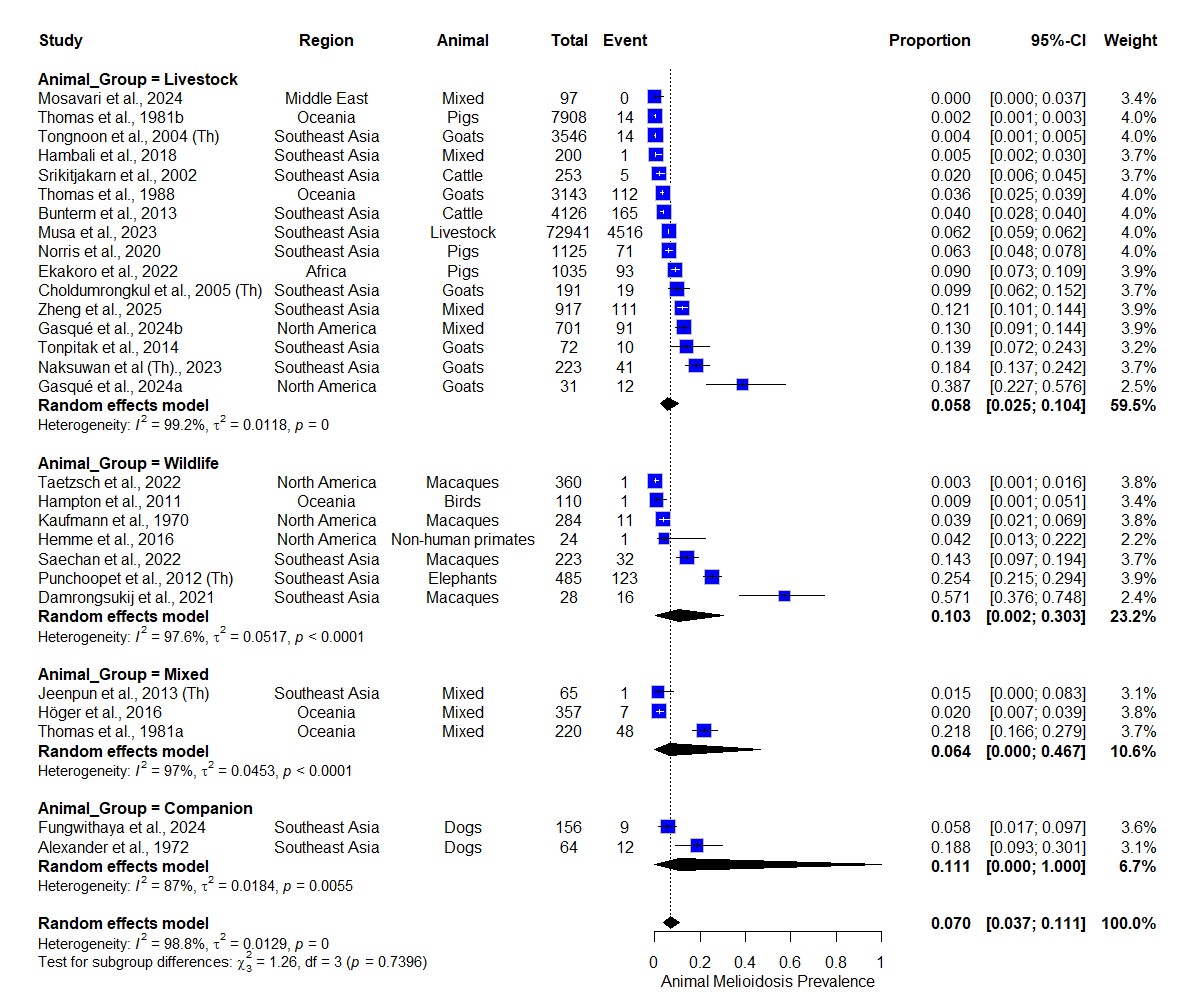

Supplement: Supplementary file 1 — Supplementary Material 1. [file 13620_2026_339_MOESM1_ESM.docx]
