# Supplementary material for: Global animal melioidosis prevalence: a systematic review and meta-analysis
Source: Ir Vet J. 2026 Mar 24;79:26. doi: 10.1186/s13620-026-00339-1 (PMC13134086; doi:10.1186/s13620-026-00339-1)
Supplement: Supplementary file 2 — Supplementary Material 2. [file 13620_2026_339_MOESM2_ESM.docx]

**Global Animal Melioidosis Prevalence: A Systematic Review and Meta-analysis**

Jongkonnee Thanasai^1^, Atthaphong Phongphithakchai^2^, Moragot Chatatikun^3,4^, Sa-ngob Laklaeng^3^, Jitbanjong Tangpong^3,4^, Pakpoom Wongyikul^5,6^, Phichayut Phinyo^5,6^, Supphachoke Khemla^7^, Anchalee Chittamma^8^, Wiyada Kwanhian Klangbud^9,*^

^1^ Faculty of Medicine, Mahasarakham University, Mahasarakham 44000, Thailand

^2^ Nephrology Unit, Division of Internal Medicine, Faculty of Medicine, Prince of Songkla University, Songkhla 90110, Thailand

^3^ School of Allied Health Sciences, Walailak University, Nakhon Si Thammarat 80160, Thailand

^4^ Research Excellence Center for Innovation and Health Products (RECIHP), Walailak University, Nakhon Si Thammarat 80160, Thailand

^5^  Center for Clinical Epidemiology and Clinical Statistics, Faculty of Medicine, Chiang Mai University, Chiang Mai 50200, Thailand

^6^ Department of Biomedical Informatics and Clinical Epidemiology (BioCE), Faculty of Medicine, Chiang Mai University, Chiang Mai 50200, Thailand

^7^ Division of Infectious Diseases, Department of Internal Medicine, Nakhon Phanom Hospital, Nakhon Phanom 48000, Thailand

^8^ Department of Pathology, Faculty of Medicine Ramathibodi Hospital, Mahidol University, Bangkok 10400, Thailand

^9^ Medical Technology Program, Faculty of Science, Nakhon Phanom University, Nakhon Phanom 48000, Thailand

***** Corresponding author: Wiyada Kwanhian Klangbud, wiyadakwanhian@gmail.com

**Supplementary Table S1.** Search strategies.

| **Search number** | **Query** | **Search Details** | **Results** | **Date** |
| --- | --- | --- | --- | --- |
| **PubMed** | | | | |
| 1 | ("melioidosis" OR "Burkholderia pseudomallei" OR "Whitmore's disease") AND ("animals" OR "livestock" OR "goats" OR "pigs" OR "cattle" OR "horses" OR "sheep" OR "monkeys" OR "fish") AND ("prevalence" OR "incidence" OR "infection rate" OR "epidemiology") | ("melioidosis"[All Fields] OR "Burkholderia pseudomallei"[All Fields] OR "Whitmore's disease"[All Fields]) AND ("animals"[All Fields] OR "livestock"[All Fields] OR "goats"[All Fields] OR "pigs"[All Fields] OR "cattle"[All Fields] OR "horses"[All Fields] OR "sheep"[All Fields] OR "monkeys"[All Fields] OR "fish"[All Fields]) AND ("prevalence"[All Fields] OR "incidence"[All Fields] OR "infection rate"[All Fields] OR "epidemiology"[All Fields]) | 186 | 05 10 2025 |
| **Embase** | | | | |
| #1 | ('melioidosis' OR 'burkholderia pseudomallei' OR 'whitmore disease') AND ('animal' OR 'livestock' OR 'goats' OR 'pigs' OR 'cattle' OR 'horses' OR 'sheep' OR 'monkeys' OR 'fish') AND ('prevalence' OR 'incidence' OR 'infection rate' OR 'epidemiology') |  | 206 | 05 10 2025 |
| **Scopus** | | | | |
| #1 | TITLE-ABS-KEY ( ( "melioidosis" OR "Burkholderia pseudomallei" OR "Whitmore's disease" ) AND ( "animals" OR "livestock" OR "goats" OR "pigs" OR "cattle" OR "horses" OR "sheep" OR "monkeys" OR "fish" ) AND ( "prevalence" OR "incidence" OR "infection rate" OR "epidemiology" ) ) |  | 204 | 05 10 2025 |
