# Supplementary material for: Global animal melioidosis prevalence: a systematic review and meta-analysis
Source: Ir Vet J. 2026 Mar 24;79:26. doi: 10.1186/s13620-026-00339-1 (PMC13134086; doi:10.1186/s13620-026-00339-1)
Supplement: Supplementary file 3 — Supplementary Material 3. [file 13620_2026_339_MOESM3_ESM.docx]

**Global Animal Melioidosis Prevalence: A Systematic Review and Meta-analysis**

Jongkonnee Thanasai^1^, Atthaphong Phongphithakchai^2^, Moragot Chatatikun^3,4^, Sa-ngob Laklaeng^3^, Jitbanjong Tangpong^3,4^, Pakpoom Wongyikul^5,6^, Phichayut Phinyo^5,6^, Supphachoke Khemla^7^, Anchalee Chittamma^8^, Wiyada Kwanhian Klangbud^9,*^

^1^ Faculty of Medicine, Mahasarakham University, Mahasarakham 44000, Thailand

^2^ Nephrology Unit, Division of Internal Medicine, Faculty of Medicine, Prince of Songkla University, Songkhla 90110, Thailand

^3^ School of Allied Health Sciences, Walailak University, Nakhon Si Thammarat 80160, Thailand

^4^ Research Excellence Center for Innovation and Health Products (RECIHP), Walailak University, Nakhon Si Thammarat 80160, Thailand

^5^  Center for Clinical Epidemiology and Clinical Statistics, Faculty of Medicine, Chiang Mai University, Chiang Mai 50200, Thailand

^6^ Department of Biomedical Informatics and Clinical Epidemiology (BioCE), Faculty of Medicine, Chiang Mai University, Chiang Mai 50200, Thailand

^7^ Division of Infectious Diseases, Department of Internal Medicine, Nakhon Phanom Hospital, Nakhon Phanom 48000, Thailand

^8^ Department of Pathology, Faculty of Medicine Ramathibodi Hospital, Mahidol University, Bangkok 10400, Thailand

^9^ Medical Technology Program, Faculty of Science, Nakhon Phanom University, Nakhon Phanom 48000, Thailand

***** Corresponding author: Wiyada Kwanhian Klangbud, wiyadakwanhian@gmail.com

**1. Meta-regression_Region**

Mixed-Effects Model (k = 28; tau^2 estimator: REML)

tau^2 (estimated amount of residual heterogeneity): 0.0333 (SE = 0.0104)

tau (square root of estimated tau^2 value): 0.1826

I^2 (residual heterogeneity / unaccounted variability): 99.38%

H^2 (unaccounted variability / sampling variability): 162.33

R^2 (amount of heterogeneity accounted for): 0.00%

Test for Residual Heterogeneity:

QE(df = 23) = 1316.1260, p-val < .0001

Test of Moderators (coefficients 2:5):

F(df1 = 4, df2 = 23) = 0.7409, p-val = 0.5739

Model Results:

estimate se tval df pval ci.lb ci.ub

intrcpt 0.3051 0.1884 1.6193 23 0.1190 -0.0847 0.6949

RegionMiddle East -0.2545 0.2711 -0.9390 23 0.3575 -0.8153 0.3062

RegionNorth America -0.0047 0.2081 -0.0227 23 0.9821 -0.4352 0.4258

RegionOceania -0.1096 0.2067 -0.5304 23 0.6009 -0.5373 0.3180

RegionSoutheast Asia 0.0095 0.1945 0.0487 23 0.9616 -0.3928 0.4118

---

Signif. codes: 0 ‘***’ 0.001 ‘**’ 0.01 ‘*’ 0.05 ‘.’ 0.1 ‘ ’ 1

**2. Meta-regression Animal group**

Mixed-Effects Model (k = 28; tau^2 estimator: REML)

tau^2 (estimated amount of residual heterogeneity): 0.0347 (SE = 0.0105)

tau (square root of estimated tau^2 value): 0.1864

I^2 (residual heterogeneity / unaccounted variability): 99.59%

H^2 (unaccounted variability / sampling variability): 243.76

R^2 (amount of heterogeneity accounted for): 0.00%

Test for Residual Heterogeneity:

QE(df = 24) = 2241.5709, p-val < .0001

Test of Moderators (coefficients 2:4):

F(df1 = 3, df2 = 24) = 0.3388, p-val = 0.7974

Model Results:

estimate se tval

intrcpt 0.3480 0.1407 2.4734

Animal_GroupLivestock -0.0934 0.1489 -0.6271

Animal_GroupMixed -0.0853 0.1808 -0.4715

Animal_GroupWildlife -0.0151 0.1598 -0.0946

df pval ci.lb

intrcpt 24 0.0209 0.0576

Animal_GroupLivestock 24 0.5365 -0.4007

Animal_GroupMixed 24 0.6415 -0.4585

Animal_GroupWildlife 24 0.9255 -0.3448

ci.ub

intrcpt 0.6385 *

Animal_GroupLivestock 0.2140

Animal_GroupMixed 0.2879

Animal_GroupWildlife 0.3146

---

Signif. codes: 0 ‘***’ 0.001 ‘**’ 0.01 ‘*’ 0.05 ‘.’ 0.1 ‘ ’ 1

**3. Meta-regression_Method**

Mixed-Effects Model (k = 28; tau^2 estimator: REML)

tau^2 (estimated amount of residual heterogeneity): 0.0336 (SE = 0.0102)

tau (square root of estimated tau^2 value): 0.1832

I^2 (residual heterogeneity / unaccounted variability): 99.34%

H^2 (unaccounted variability / sampling variability): 151.32

R^2 (amount of heterogeneity accounted for): 0.00%

Test for Residual Heterogeneity:

QE(df = 24) = 1266.6116, p-val < .0001

Test of Moderators (coefficients 2:4):

F(df1 = 3, df2 = 24) = 0.5395, p-val = 0.6598

Model Results:

estimate se tval df pval ci.lb

intrcpt 0.2941 0.0865 3.4000 24 0.0024 0.1156

Method_typeMixed -0.0444 0.1303 -0.3409 24 0.7362 -0.3133

Method_typeMolecular -0.2435 0.2144 -1.1358 24 0.2672 -0.6860

Method_typeSerology 0.0022 0.0978 0.0229 24 0.9819 -0.1997

ci.ub

intrcpt 0.4727 **

Method_typeMixed 0.2245

Method_typeMolecular 0.1990

Method_typeSerology 0.2042

---

Signif. codes: 0 ‘***’ 0.001 ‘**’ 0.01 ‘*’ 0.05 ‘.’ 0.1 ‘ ’ 1

**4. Meta-regression_Peroid**

Mixed-Effects Model (k = 28; tau^2 estimator: REML)

tau^2 (estimated amount of residual heterogeneity): 0.0322 (SE = 0.0098)

tau (square root of estimated tau^2 value): 0.1795

I^2 (residual heterogeneity / unaccounted variability): 98.93%

H^2 (unaccounted variability / sampling variability): 93.56

R^2 (amount of heterogeneity accounted for): 0.00%

Test for Residual Heterogeneity:

QE(df = 24) = 936.3473, p-val < .0001

Test of Moderators (coefficients 2:4):

F(df1 = 3, df2 = 24) = 1.0022, p-val = 0.4089

Model Results:

estimate se tval df pval ci.lb ci.ub

intrcpt 0.2704 0.0836 3.2332 24 0.0035 0.0978 0.4430

Period2000-2010 -0.0933 0.1361 -0.6856 24 0.4995 -0.3742 0.1876

Period2011-2020 -0.0354 0.1049 -0.3374 24 0.7387 -0.2520 0.1812

Period2021-2025 0.0812 0.1013 0.8017 24 0.4306 -0.1278 0.2902

intrcpt **

Period2000-2010

Period2011-2020

Period2021-2025

---

Signif. codes: 0 ‘***’ 0.001 ‘**’ 0.01 ‘*’ 0.05 ‘.’ 0.1 ‘ ’ 1
