# Supplementary material for: Global animal melioidosis prevalence: a systematic review and meta-analysis
Source: Ir Vet J. 2026 Mar 24;79:26. doi: 10.1186/s13620-026-00339-1 (PMC13134086; doi:10.1186/s13620-026-00339-1)
Supplement: Supplementary file 5 — Supplementary Material 5. [file 13620_2026_339_MOESM5_ESM.docx]

**Global Animal Melioidosis Prevalence: A Systematic Review and Meta-analysis**

Jongkonnee Thanasai^1^, Atthaphong Phongphithakchai^2^, Moragot Chatatikun^3,4^, Sa-ngob Laklaeng^3^, Jitbanjong Tangpong^3,4^, Pakpoom Wongyikul^5,6^, Phichayut Phinyo^5,6^, Supphachoke Khemla^7^, Anchalee Chittamma^8^, Wiyada Kwanhian Klangbud^9,*^

^1^ Faculty of Medicine, Mahasarakham University, Mahasarakham 44000, Thailand

^2^ Nephrology Unit, Division of Internal Medicine, Faculty of Medicine, Prince of Songkla University, Songkhla 90110, Thailand

^3^ School of Allied Health Sciences, Walailak University, Nakhon Si Thammarat 80160, Thailand

^4^ Research Excellence Center for Innovation and Health Products (RECIHP), Walailak University, Nakhon Si Thammarat 80160, Thailand

^5^  Center for Clinical Epidemiology and Clinical Statistics, Faculty of Medicine, Chiang Mai University, Chiang Mai 50200, Thailand

^6^ Department of Biomedical Informatics and Clinical Epidemiology (BioCE), Faculty of Medicine, Chiang Mai University, Chiang Mai 50200, Thailand

^7^ Division of Infectious Diseases, Department of Internal Medicine, Nakhon Phanom Hospital, Nakhon Phanom 48000, Thailand

^8^ Department of Pathology, Faculty of Medicine Ramathibodi Hospital, Mahidol University, Bangkok 10400, Thailand

^9^ Medical Technology Program, Faculty of Science, Nakhon Phanom University, Nakhon Phanom 48000, Thailand

***** Corresponding author: Wiyada Kwanhian Klangbud, wiyadakwanhian@gmail.com

# Supplementary Table S2. JBI Critical Appraisal Checklist for Prevalence Studies

| **No.** | **Study, Year** | **Country / Species** | **JBI Score (Total 9)** | **Quality** | **Key Strengths** | **Main Limitations** |
| --- | --- | --- | --- | --- | --- | --- |
| 1 | Alexander et al., 1972 | Vietnam / Military dogs | 6/9 | Moderate | Clear population and laboratory-based IHA test; comparison before-after exposure. | Convenience sampling, limited representativeness, no CI reported. |
| 2 | Bunterm & Bunterm, 2013 (Th) | Thailand / Dairy cattle | 8/9 | High | Large sample (n=4,126), IHA standardized, multi-year, risk factors analyzed. | Some retrospective data, limited randomization of farms. |
| 3 | Choldumrongkul, 2005 (Th) | Thailand / Elephants (captive) | 7/9 | High | Clear criteria, validated diagnostic test (IHA), defined study period. | Small sample size, no confidence intervals reported. |
| 4 | Damrongsukij et al., 2021 | Thailand / Livestock serology | 8/9 | High | Representative national sampling, validated test (ELISA/IHA), transparent methods. | Potential recall bias in farmer data. |
| 5 | Ekakoro et al., 2022 | Uganda / Goats and cattle | 7/9 | High | Randomized sampling, ethical approval, modern diagnostics. | Possible underestimation due to single timepoint sampling. |
| 6 | Fungwithaya et al., 2024 | Thailand / Dogs (shelter) | 8/9 | High | Clear sampling (n=156), ethics approved, validated IHA, and correlation analysis. | Shelter-only dogs, limited generalizability. |
| 7 | Gasqué et al., 2024 | Guadeloupe & French Guiana / Livestock | 9/9 | High | Representative design, validated ELISA, environmental confirmation, longitudinal data. | None major – well designed. |
| 8 | Gasqué et al., 2024 (2) | Guadeloupe / Goat longitudinal | 9/9 | High | Longitudinal design, paired serology, environment linkage. | None major. |
| 9 | Hambali et al., 2018 | Malaysia / Sheep & goats | 7/9 | High | CFT standardized, ethical, clear methodology, results quantifiable. | Small sample, single positive case, low power. |
| 10 | Hampton et al., 2011 | Australia / Wild & captive birds | 6/9 | Moderate | Field surveillance, molecular confirmation, diverse species. | Opportunistic sampling, no prevalence CI, small sample for wild birds. |
| 11 | Hemme et al., 2016 | Guadeloupe / Livestock | 8/9 | High | Good sample frame, validated ELISA. | Cross-sectional, no CI in subgroups. |
| 12 | Höger et al., 2016 | Madagascar / Goats, pigs | 7/9 | High | Environmental mapping, PCR. | Incomplete animal data. |
| 13 | Jeenpun et al., 2013 (Th) | Thailand / Livestock | 7/9 | High | IHA standardization. | Limited farm distribution. |
| 14 | Kaufmann et al., 1970 | Thailand / Equines | 6/9 | Moderate | Classic reference, culture isolation. | Very small N, historic bias. |
| 15 | Mosavari et al., 2024 | Iran / Ruminants | 8/9 | High | Abattoir-based One Health sampling, PCR + biochemical confirmation. | Non-random lesions-only sampling. |
| 16 | Musa et al., 2023 | Malaysia / Livestock (multi-species) | 9/9 | High | Massive dataset (72,941), CFT validated, correlation analysis. | Retrospective 10-year dataset. |
| 17 | Naksuwan et al., 2003 (Th) | Thailand / Goats | 8/9 | High | 10-year pathology + culture confirmation, risk factor analysis. | Retrospective necropsy data only. |
| 18 | Norris et al., 2020 | Vietnam / Swine | 9/9 | High | ELISA multi-antigen validation, large sample (n=1,125). | No temporal replication. |
| 19 | Punchoopet et al., 2012 (Th) | Thailand / Elephants | 8/9 | High | Nationwide IHA survey (n=485). | Lack of CI, small southern data. |
| 20 | Saechan et al., 2022 | Thailand / Wild macaques | 8/9 | High | Field study, IHA + PCR verification. | Low positivity (14%), limited sites. |
| 21 | Srikitjakarn et al., 2002 | Thailand/ Cattle | 7/9 | High | IHA standardization | Low cut off IHA (> 40) |
| 22 | Taetzsch et al., 2022 | USA / Domestic animals (imported) | 7/9 | High | Clear epidemiological tracing, PCR confirmation, CDC dataset. | Passive surveillance, imported bias. |
| 23 | Thomas et al., 1981 | Australia / Pigs | 7/9 | High | Abattoir-based, bacteriological confirmation, 5-year dataset. | No random sampling, small N per year. |
| 24 | Thomas et al., 1988 | Australia / Pigs & environment | 8/9 | High | Longitudinal data, culture confirmation, water–soil linkage. | No CI reported, older methodology. |
| 25 | Tongnoon, 2004 (Th) | Thailand / Livestock (multi-species) | 8/9 | High | IHA validated, clear test cutoff (1:160), multiple provinces. | Limited randomization, descriptive. |
| 26 | Tonpitak et al., 2014 | Thailand / Dairy cattle | 9/9 | High | National random survey, ELISA validated, spatial analysis. | None major – strong design. |
| 27 | Zheng et al., 2025 | Lao PDR / Buffalo, cattle, swine | 9/9 | High | Large sample (n=917), systematic sampling, GIS mapping. | IHA-only limitation, no ELISA. |
| 28 | Thomas et al., 1981 (ver.1) | Australia / Pigs | 6/9 | Moderate | Field bacteriology, early endemic confirmation. | Convenience sampling, no CI, incomplete metadata. |

| Criterion |
| --- |
| 1. Sample frame appropriate to address target population |
| 2. Study participants sampled appropriately |
| 3. Adequate sample size |
| 4. Study subjects described in detail |
| 5. Valid methods used for identification of condition |
| 6. Standard measurement for all participants |
| 7. Appropriate statistical analysis |
| 8. Adequate response rate & data coverage |
| 9. Clear reporting of results and CIs |
